# Navigating Challenges in Peer Support Work: Perspectives of Peer Supporters From a Stepped Care Intervention for Older Adults With Depressive Symptoms

**DOI:** 10.1111/hex.70430

**Published:** 2025-09-10

**Authors:** Eric Kwok Lun Yiu, Stephanie Ming Yin Wong, Jessica Kang Qi Lee, Zuna Loong Yee Ng, Dara Kiu Yi Leung, Wai Chi Chan, Gloria Hoi Yan Wong, Terry Yat Sang Lum

**Affiliations:** ^1^ Department of Social Work and Social Administration The University of Hong Kong Hong Kong China; ^2^ Department of Social Work The Chinese University of Hong Kong Hong Kong China; ^3^ Department of Psychiatry, School of Clinical Medicine, LKS Faculty of Medicine The University of Hong Kong Hong Kong China; ^4^ School of Psychology and Clinical Language Sciences University of Reading Reading UK

**Keywords:** depression, healthy ageing, mental health services, peer support, productive ageing

## Abstract

**Background:**

Serving as peer supporters in later life has been linked to a greater sense of purpose and meaning in life. How the wisdom of older adults could be leveraged to improve the implementation of peer support work, however, has rarely been considered. We aimed to examine the perspectives of peer supporters in this study, including the challenges they encountered in practice and the strategies they developed to navigate their roles.

**Methods:**

A series of semi‐structured focus group interviews was conducted with 23 peer supporters (mean age = 66.2 years, SD = 7.1) in a community‐based stepped‐care intervention service for depressive symptoms in older adults aged 60 years and older in Hong Kong. Data were analysed using inductive thematic analysis and discussed with peer supporters to ensure relevance.

**Results:**

Three themes emerged to reflect challenges faced in peer support work: role ambiguity, boundary setting, and engaging with service users. To address these challenges, centring attention on meaningful engagement with users, managing expectations of users regarding the scope of peer support, and expanding the social networks of both users and peer supporters were prioritised. The importance of professional development through continuous learning and mentorship programmes, as well as creating user‐centred environments, was also highlighted.

**Conclusions and Implications:**

These findings suggest that older adults have the potential to actively navigate through service challenges by leveraging their internal capabilities and external resources. Integrating these insights into future peer support service design could enhance service delivery and empower older adults to become active agents of employment and care, thereby contributing to productive and healthy ageing initiatives.

**Patient and Public Contribution:**

The JoyAge Peer Support network adopts a co‐production and partnership approach to designing all services and research. The present work was part of an effort to ensure that the lived experiences and wisdom of peer supporters could be reflected in future service design. All findings of this study were discussed with peer supporters to ensure their relevance.

## 1 Background

By 2030, one in six people will be aged 60 years or older [1], with the average life expectancy at age 65 projected to be around 20 years [2, 3]. To address the impacts of rapid population ageing and increasing life expectancy on the shrinking working‐age population, the active participation of older adults in the labour force can be crucial [4, 5]. Continued employment in meaningful capacities in late life not only has implications at societal and economic levels but can also provide older adults with opportunities to live with a greater sense of purpose and dignity, contributing to enhanced well‐being and ‘ageing well’ initiatives [6, 7, 8].

A promising means of scaling up meaningful employment opportunities in later life is through peer support. Peer support work, which typically involves training people with shared life experiences in the provision of care, has increasingly been adopted in mental healthcare [9, 10]. Despite being traditionally considered in the context of severe mental illness, evidence has shown the potential of expanding peer support services to the older adult population to foster productive ageing [7, 11]. From this perspective, older adults are no longer passive recipients of care [12] but rather individuals who possess the capabilities to contribute to their local communities through active participation in economic and social activities, and in turn, reduce the strain on the healthcare system [13]. By leveraging their personal experiences to support others facing similar challenges related to ageing and mental health distress, the involvement of peer support has been shown to facilitate the recovery of service users by reducing stigma and power imbalances, while inducing a greater sense of hope [9, 14]. Notably, previous work has also demonstrated positive impacts on older adults who serve as peer supporters (e.g., increased sense of meaning and companionship) [7, 15].

Despite the potential benefits of building capacity among older adults in peer support, several challenges have also been identified in practice. For instance, feelings of knowledge deficits, uncertainties about roles and responsibilities, and insufficient financial compensation have been reported among peer supporters [16, 17, 18]. Inadequate supervision and limited autonomy in supporting service users have also been considered as barriers [16, 19]. However, as also highlighted in a recent study of peer supporters from a diverse range of contexts, the existing knowledge base has predominantly been developed through the viewpoints of service users or organisations [19]. Current strategies designed to overcome these challenges have also been based on the perspectives of professionals (e.g., implementing more rigorous selection criteria for peer supporters and developing service manuals) rather than those of peer supporters themselves [4, 5, 16]. It remains unclear what challenges peer supporters might face in the context of late‐life mental health services, and more importantly, what strategies they have developed to facilitate them in navigating through these challenges in the real‐world setting.

Of note, Hong Kong is one of the fastest ageing regions in the world with a leading old‐age dependency ratio [20]. The local government has projected a substantial rise in the proportion of older adults aged 65 years or older, from 1.52 million in 2021 to 2.95 million in 2046—namely, from 20.5% to 30.6% of the population [21]. The employment rate of older adults in Hong Kong, however, is relatively lower than that in other East Asian economies [22], with workplace discrimination often encountered in this age group [23]. Further, while peer support services are well‐developed in Western countries, peer support in geriatric mental health services is a relatively new phenomenon in non‐Western societies [7]. Most existing peer support services for mental health, particularly in Asia, are also targeted at young and middle‐aged adults [24]. To date, very few studies have examined the experiences of older adults engaging in non‐Western peer support services for late‐life depression. To alleviate the problem of mental health manpower shortage and promote productive ageing, understanding the potential challenges encountered by older adults trained as mental health peer supporters in Hong Kong may serve as an important reference for other rapidly ageing populations.

Through a series of focus group interviews, the present study thus aimed to uncover the challenges encountered by older adults in their peer support roles within a collaborative stepped‐care intervention service for late‐life depression, as well as the strategies they utilised to navigate these. Understanding their first‐hand perspectives is crucial to improving future service and policy developments in peer support work and mental healthcare. By considering these strategies as solutions to possible implementation barriers, older adults can be honoured as major contributors to the development of successful peer support work for geriatric mental health services in the context of productive and healthy ageing.

## 2 Methods

### 2.1 Study Design

An exploratory qualitative approach was employed to facilitate a deeper and more nuanced understanding of the experiences of peer support provision among older adults within both a recovery and a productive ageing framework. The focus group method was chosen to stimulate dynamic discussions among different peer supporters, thereby allowing for the emergence of diverse viewpoints and collective insights [25].

### 2.2 Participants

Trained peer supporters were recruited from Phase II of the Jockey Club Holistic Support Project for Elderly Mental Wellness (JC JoyAge–2) from July to October 2023. JoyAge is a territory‐wide, community‐based, free‐of‐cost collaborative stepped‐care intervention project for older adults aged 60 years or above with mild to moderately severe depressive symptoms in Hong Kong. Cognitive behavioural therapy‐based interventions were the core service modality, provided by trained social workers under the supervision of clinical psychologists and assisted by peer supporters. Since its first implementation in 2016 across four districts (Phase I), the service model has expanded to 12 districts from 2020 to 2021, and subsequently to all 18 districts of Hong Kong since 2022. During its second phase, JoyAge services were provided in 47 district elderly community centres and integrated mental wellness centres (which have now been further expanded to 51 service units in the new project phase since 2024, including in the primary care setting). Details of the project have been reported elsewhere [7, 26, 27].

All peer supporters received an intensive series of training (80 h total), covering knowledge of geriatric mental health, the recovery model, peer support skills and knowledge, mental health resources, and self‐care, as well as clinical practicum. In an effort to foster meaningful engagement in the workforce in later life, all peer supporters of JoyAge were officially employed on an hourly basis at salary rates exceeding the local minimum wage, with slight variations across service units (approximately USD$8.75 to USD$9.38 per hour). Participants were included if they were (1) aged 50 years and older, (2) completed the 80‐h JoyAge peer supporter training involving both theory and practicum, (3) currently employed by service units of the project, and (4) able to provide informed consent. At the time of data collection, the project had trained over 829 peer supporters. Purposive sampling was employed with consideration of participant sex, age, mental health history, years of service as a peer supporter, and number of service users supported to ensure a more diverse range of perspectives could be captured.

Interested peer supporters were invited to complete an online registration survey with the above information and their available time slots. Initially, 21 participants from 10 service units showed interest, although only four of them reported a history of major depression. A second round of recruitment was thus conducted to target peer supporters with a history of major depression, resulting in four additional participants. After excluding two participants who withdrew due to urgent personal obligations, the final study sample included 23 participants across 11 service units.

### 2.3 Procedures

Five face‐to‐face focus groups were conducted in Cantonese, each comprising 3–6 participants with varying socio‐demographics and experience in service provision. Each focus group lasted 90–120 min, with a 15‐min break, and was facilitated by at least two researchers trained in psychology or social work (E.K.L.Y., H.H.L.C., M.W.S.L. or H.S.K.). After consenting, participants were invited to complete a survey that assessed their perceived self‐competence in providing peer support services on five domains selected based on our previous Delphi study (i.e., self‐care, work ethics, teamwork, working with service users, and peer support knowledge) [28]. The focus groups were guided by a semi‐structured interview and informed by responses from the competence survey (e.g., ‘Most of you rated work ethics as the weakest competency. What was the reason behind this?’) (Table S2). The interview guidebook was co‐developed by two clinical psychologists (W.W.K. and A.K.Y.S.), one senior researcher (T.Y.L.) and four researchers (E.K.L.Y., H.H.L.C., M.W.S.L. and H.S.K.) who worked closely with peer supporters in the project to ensure their clinical relevance.

Apart from the two clinical psychologists, the researchers who conducted the focus group interviews did not provide any direct intervention to JoyAge users nor training to peer supporters. While researchers of the team saw the value in peer support, the main goal of the study was to reflect the real‐world experiences of peer supporters. The research team was thus conscious of not directing participants to respond in favour of peer support (e.g., no guiding questions were asked). The guidebook was also developed through a reflexive process.

Following the first focus group, the interview questions were refined based on feedback from participants and interviewers. In the original guidebook, a question concerning peer supporter training was initially asked to gauge participants' recall of training (‘Do you remember the training materials or theory?’), which was followed by questions concerning whether the training had facilitated their management of service challenges. Nevertheless, most interviewees in the first group could not pinpoint details from the training. We therefore rearranged the questions and began the focus groups by asking about service challenges they encountered in practice, and replaced the question on training details with another related to the perceived effectiveness of training (i.e., ‘Do you think the training has been sufficient in facilitating your service provision?’). Also, two service‐related questions (i.e., ‘*How would you introduce the Peer Supporter role to service users?’* and *‘How would you describe your relationships with service users and social workers?’*) were added to the beginning of the focus groups as prompts to facilitate experience sharing. All interviewers were involved in refining the questions and followed this modified interview guidebook in the remaining focus groups.

### 2.4 Data Analysis

All focus groups were audio‐recorded with consent from each participant. The recordings were transcribed anonymously, with verbatim analysis applied using NVivo following inductive reflexive thematic analysis [29]. This approach allowed researchers to openly explore potential themes based on our data. Since the original verbatim was in Cantonese, two researchers (E.K.L.Y. and J.K.Q.L.) were involved in the translation process. The verbatim was first translated into English and presented using culturally appropriate pseudonyms (led by J.K.Q.L.). Another researcher (E.K.L.Y.) then independently reviewed the translations to ensure they reflected the meaning of the original quotes. Both researchers were fluent in the languages (since both Chinese and English are official languages in Hong Kong) and had experience in translation procedures in previous qualitative studies. To ensure anonymity and confidentiality, only de‐identified data were presented in this manuscript.

Following standard procedures [30], a six‐stage process was applied for the thematic analysis: (1) three researchers trained in psychology (E.K.L.Y., Z.L.Y.N. and J.K.Q.L.) first familiarised themselves with the data and independently read the transcripts of the first three focus groups; (2) based on narratives surrounding peer supporters' challenges and strategies, the three researchers separately generated initial codes. Together with a senior researcher (S.M.Y.W.), the team co‐developed a coding framework for application to the entire dataset; (3) the research team identified possible themes based on the coded data; (4) the three researchers revisited the coded data to ensure their congruence with the themes, as well as relevance and accuracy of identified themes. The remaining transcripts were also coded based on the identified themes; (5) each theme was then derived and named following consensus among the team; and (6) the finalised themes were then reported in this manuscript, supported by verbatim of participants and with reference to the relevant literature.

To enhance the trustworthiness and credibility of the findings, all interviewed peer supporters were contacted to review the finalised themes and determine whether they accurately reflected their experiences in peer support. Most affirmed their accuracy, with minor feedback received to enrich examples of the identified themes, which were refined and presented in this manuscript. De‐identified participant number, sex and age were indicated for each quote. The results of this present study were reported in accordance with the Standards for Reporting Qualitative Research [31] (Table S1).

### 2.5 Ethical Approval

All participants provided informed consent for the study and retention of their focus group recordings. The study was approved by the Human Research Ethics Committee of The University of Hong Kong (reference no. EA2003001).

## 3 Results

Among the 23 peer supporters, their mean age was 66.2 years; the majority were female (73.9%). Seven of them self‐reported a history of major depressive disorder. Twenty‐one were employed in district‐based elderly community centres, and two were employed in integrated community centres for mental wellness. Their average employment period was 2.4 years (SD = 1.8), with 20 peer supporters (87%) having provided service in their respective service units for over a year. Each peer supporter interviewed provided service to an average of six service users. Details of their socio‐demographics are shown in Table 1.

**Table 1:** Characteristics of peer supporters interviewed (*n* = 23).

	*n* (%)/mean (SD)
Sex	
Female	17 (73.9%)
Male	6 (26.1%)
Age, mean (SD)	66.2 (7.1)
History of a major depressive disorder diagnosis	7 (30.4%)
Service unit	
District elderly community centre	21 (91.3%)
Integrated mental wellness centre	2 (8.7%)
Service period (in years)	2.37 (1.75)
< 1	3 (13%)
1–2	11 (47.8%)
≥ 3	9 (39.1%)
Number of followed service users (mean, SD)	6.1 (5.6)

*Note:* All values are presented in the form of *n* (%) unless otherwise stated.

Three overarching themes related to the challenges encountered by peer supporters in their service provision emerged: (1) ambiguity on peer support status; (2) maintaining boundaries with service users; and (3) engaging with service users effectively. Corresponding to these, six core peer support strategies were identified: (1) centring attention on meaningful engagement and recognition; (2) managing expectations and drawing consensus; (3) building supportive networks and alternative resources; (4) proactive learning and continuous development; (5) creating a safe and user‐centred environment; and (6) fostering collaboration through peer sharing and mentorship (Table 2). An additional strategy raised by peer supporters was supervision by social workers to guide and monitor their progress in handling those challenges. All identified themes were reviewed, with the selection of quotes refined following comments from the peer supporters interviewed.

**Table 2:** Thematic framework of the challenges and strategies to peer support service provision.

Challenges	Strategies adopted
1. Ambiguity on peer support status—volunteering or employment?	Centring attention on authentic and meaningful engagement with service users
2. Maintaining boundaries with service users	Managing expectations and drawing a consensus Building support networks (both service users and peer supporters) and working collaboratively
3. Engaging with service users	Proactive learning and continuous development Fostering collaboration through peer sharing and mentorship Creating a safe and user‐centred environment

### 3.1 Ambiguity on Peer Support Status—Volunteering or Employment?

While peer supporters in the current mental health service were employed to provide support on an hourly basis, most peer supporters considered themselves volunteers with subsidies. They explained that their initial intention of working as peer supporters was not driven by financial reasons, but rather contribute to the community during their retirement stage. One peer supporter shared feeling lost and uncertain as to whether he should see himself as an employee, a volunteer, or a friend of service users:

The emotional journey of being a Peer Supporter is becoming increasingly complex. Initially, I saw it as just volunteering, and through that experience, I made some great friends. However, over time, I began to receive compensation for my efforts. Now, all these feelings are intertwined.

PS15, Male, Age 53

Although the peer supporters found meaning in their roles, some shared an increasing amount of pressure related to their work duties imposed upon them (e.g., required service hours per month). One reported feeling overwhelmed and worried:

The pressure from the contract is that you have to work 17 h every month. It really depends on the clients. For me, my clients aren't particularly demanding, so I don't want to bother them too much, and I don't contact them often. Then I start worrying, “Oh no, I haven't met the hours this month.” Sometimes I feel a bit down, wondering why I didn't meet the target again this month.

PS02, Female, Age 59

Some peer supporters also noted that, while they are aware of the importance of ‘walking together’ with others facing mental health challenges, service users may not be familiar with terms such as ‘peer supporter’ and ‘peer support’. Many, therefore, chose to introduce themselves as volunteers instead:

As a volunteer, I realised that not all clients fully understand our role. When the social worker connected us, some clients may not have grasped that we are here to walk alongside them in their journey. While many of them do understand that they can reach out to us when they're feeling down or need support, others might initially focus on questions about administrative procedures related to social welfare.

PS01, Female, Age 52

Nevertheless, some peer supporters were also clear about the differences between peer support and regular volunteering services:

Volunteering is different from being a peer supporter. […] Peer supporters are directly engaged with individuals, focusing on their emotional and psychological well‐being. They work closely with clients to provide tailored support and guidance based on their needs. Volunteer work doesn't involve case management; it mainly consists of participating in large events or assisting with activities at the centre.

PS22, Male, Age 67

#### 3.1.1 Strategy 1: Centring Attention on Authentic and Meaningful Engagement

To address the ambiguity between employment and volunteering, peer supporters shared their experiences of shifting their focus from the sense of uncertainty surrounding their titles, salaries or working hours to engaging meaningfully with service users. One noted that the peer support service represents just one of the various opportunities and shared an example:

Our initial goal wasn't about making money; we were focused on connecting with people. […] I found that I was putting in a lot of effort into preparation—more than just standing around doing tasks. Often, even when two hours are up, if there's still a need to talk or listen, we stay engaged. It's definitely not a matter of looking at the clock and saying, “Okay, it's been two hours, time to leave.” That's just not how it works.

PS13, Female, Age 69

Another peer supporter also shared her approach to viewing peer support work as a joyful and rewarding experience to overcome the pressure of fulfilling service hours:

It (Working as a Peer Supporter) means I can prove to my children that their mother is capable—it's not that I can't do anything. Although I've never worked outside of the home before, I'm now contributing in this role—look, I even have my own pocket money. Isn't that something to feel happy about? I won't stress myself out. If I don't reach 16 h, then I don't. If I exceed 16 h, I'll note it in the document. It just shows that I am doing something. I have options now. I won't put pressure on myself. […] If I enjoy it, I do it. If I don't, I don't.

PS13, Female, Age 69

Further, some considered it helpful to directly clarify their roles and involvements with their service users:

I told her that I needed to explain my role in her life. I sensed some resistance from her, so I wanted to clarify who I was and why I was involved in her situation.

PS16, Female, Age 68

### 3.2 Maintaining Boundaries With Service Users

Unlike traditional full‐time employment, these peer supporters often had more flexible working hours and less stringent duty reporting procedures. The strategy of emphasising meaningful engagement with service users may, however, bring another layer of challenge. For instance, this job flexibility may lead peer supporters to engage with service users outside of regular work hours, including those who have been discharged from the service. One peer supporter specifically highlighted that the unlimited overtime could feel overwhelming in the long run:

Sometimes, we find ourselves doing things that don't pay off. […] I often tell people (service users) that I'm really busy, and they respond by saying I can get back to them when I have time. However, they keep messaging repeatedly, and before you know it, there are so many messages piled up that you feel obligated to respond. The problem is, there's no financial reward for replying to these clients who have already been discharged. Even when it comes time to meet with them, there's no money involved. So it's not just about giving up seventy dollars; it's about the time and effort we invest without any compensation.

PS21, Female, Age 65

Another peer supporter shared that, despite the opportunity to build emotional connections with the service users, it could also complicate their ability to set boundaries outside working hours:

I often find myself wanting to say, “Look, we're just here to work. Once our hours are up, I won't be thinking about you.” But honestly, it's tough to express that, and I'm not sure how to manage the situation. […] There's one client who still checks in on me regularly, asking how I'm doing. This usually means they want me to visit them again. Sometimes, I can't help but feel a bit heartless for wanting to keep my distance.

PS13, Female, Age 69

Frustrations may also be experienced when the boundaries with service users cannot be clearly set:

I feel like a failure, and I've been labelled as one. From the very beginning, the social worker advised us not to share our phone numbers or spend too much time with the individuals we were helping, as it could complicate our lives. But my mistake was that I ended up giving them my phone number anyway. […] Honestly, I'm not sure how to handle this situation. […] When they asked for my number, I was torn between wanting to support them and knowing that sharing my contact information could lead to complications. It's a dilemma I'm still grappling with.

PS13, Female, Age 69

#### 3.2.1 Strategy 1: Managing Expectations and Drawing Consensus

Upon facing issues related to boundary setting with service users, some shared their approach to setting clearer definitions in their relationships at the outset. This was considered helpful in managing the expectations of service users and facilitated their work:

When you're starting out as a new Peer Supporter or taking on a new client, it's essential to establish clear boundaries from the very beginning. You might say something like, “I won't be sharing my personal phone number, and I won't create a WhatsApp group for us. I'll let you know how and when we can stay in touch.” Being upfront about these boundaries helps the person understand the nature of your relationship and sets the expectation that there may be times when you're not available. It's crucial to communicate these points early on so that everyone is on the same page. This clarity helps prevent complications later if you need to step back.

PS12, Male, Age 61

Another peer supporter also shared that if there were occasions in which additional assistance or support was required beyond working hours, their roles should be clearly clarified with the service user:

I have my own boundaries, and I need to explain to him why I was willing to help him the first time—it just so happened that I was free. This means I won't be able to do it again. I want to be clear about this from the beginning. It's important for him to understand that we are partners on this journey; I'm not just here to support him or be his crutch. We are all in this together.

PS18, Female, Age 70

When it comes to receiving gifts or benefits from service users, a peer supporter also shared the importance of establishing a consensus:

I often go for yum cha (Chinese brunch) with the older person, but I always make it clear that we split the bill. They frequently offer to treat me, but I explain that I can't accept it. I tell them that while I can treat them or give them gifts, they cannot give me anything in return. That's just how our rules work. So when we go for yum cha, we always share the cost. It's essential to me that I decline any gifts or benefits from them. They often ask, “What can I give you?” and I simply say that I won't accept anything.

PS19, Female, Age 73

#### 3.2.2 Strategy 2. Building Support Networks and Working Collaboratively

Apart from explicitly setting clear boundaries, the peer supporters also noted the importance of building support networks, both among service users and peer supporters themselves. For example, one peer supporter shared:

We can connect one person with another so they can support each other after they've opened up to us. Since we all live in the same area, it's really beneficial for them to have someone to talk to, rather than relying solely on us. […] But if two or three people can form a support circle and provide emotional support to each other, it allows us to concentrate on helping even more people.

PS17, Female, Age 66

The peer supporters were also aware of the limitations of working alone and reminded themselves to seek support from fellow peers when situations were beyond their control and availability:

It doesn't always have to be me handling everything. We all have our own busy schedules and responsibilities. That's why other Peer Supporters can jump in to help out. We support each other, so it's not like one person is left to manage all the clients alone.

PS02, Female, Age 59

Another peer supporter noted the importance of being aware of their own abilities and seeking support from professionals when needed:

If we can't manage the situation, it's best not to push it. We'll simply pass the cilent back to the social workers as they're the experts in this area, and we're just on the front lines. You're doing the right thing by recognising that. When it's beyond our control, forcing it won't lead to a positive outcome. It's really better to let them take charge and handle it.

PS23, Female, Age 70

### 3.3 Engaging With Service Users Effectively

As the majority of the peer supporters interviewed had been in service for over a year and had completed their training over 2 years before the present study, many reported finding it difficult to recall the materials and theories learned related to peer support services and skills. As such, they predominantly relied on their own experiences when engaging service users. One peer supporter shared difficulties in responding to service users on the spot:

…yeah, I can't always recall things on the spot. After a call, I often think, “I should have said it this way.” I have all these thoughts in my head, but they don't always come to me when I need them.

PS14, Female, Age 69

Engaging with service users undergoing major life stressors, such as grief and bereavement, can be even more challenging:

For instance, if someone has recently lost a spouse, it's best to avoid bringing that up. So, what topics can you use to connect with them? From our observations, we've noticed that awkward silences can often occur, leaving both parties unsure of what to say next—dead air.

PS12, Male, Age 61

Notably, both male and female peer supporters reported challenges in supporting service users of the opposite gender or sex. One female peer supporter shared that she had to be careful when chatting with male service users and avoid potential misinterpretations of her caring actions:

But I have to say, taking care of the opposite sex can be a bit tricky. Sometimes, if you show too much concern, it can be misunderstood. […] I have a male client, and our interactions can sometimes feel awkward. He prefers that I don't visit him at home, so I invite him to come to the centre for a chat instead. However, he has his own friends, and he seems embarrassed to pick up the call from the opposite sex in front of them.

PS09, Female, Age 66

Meanwhile, a male peer supporter also commented that the centres' activities often attracted more female than male participants. It can then become quite difficult for him to develop the skills to involve male service users in services:

…there are very few male participants, no matter where I go. This often leads to the centre's activities being more geared towards women, like crafts or artistic pursuits. […] Unfortunately, these types of activities might not be enough to keep the male members engaged. […] Since there are so few male cases, after a couple of years in this role, I'm starting to run out of male service users to support, which is becoming an issue. I may need to start taking on female service users, which would be more challenging for me.

PS06, Male, Age 60

#### 3.3.1 Strategy 1: Proactive Learning and Continuous Development

The peer supporters agreed that even after undergoing in‐depth training, it does not mean they remember all the skills learned and apply them all the time. Therefore, continued learning and discussions with other peer supporters and social workers can be crucial. One peer supporter highlighted the importance of reviewing training notes:

I often refer to those notes because sometimes you just get stuck on something. […] For instance, that stack of materials you gave me—whether it's practical or theoretical—acts like a dictionary for me. I find myself looking things up in it from time to time. If anyone tells you that you can absorb everything during the course, they're definitely not being truthful.

PS01, Female, Age 52

They also showed proactiveness in learning new knowledge to improve peer support services:

From my perspective, I believe it's about doing our best. I tend to take things as they come. If I have the time and relevant training sessions are available, I will definitely attend them. This way, I gradually learn more and more.

PS14, Female, Age 69

Another peer supporter shared a similar view:

That's right, we need to focus on improving ourselves. It's essential for us to take the initiative in learning about what's happening in the community. For instance, many older adults struggle with using smartphones or finding their way around. If you can teach them even a little, it can make a huge difference compared to not being able to help at all. […] By understanding the needs of your clients, you can help them better integrate into the community.

PS09, Female, Age 66

A few also suggested that local universities may offer practical mental health courses for older adults, with cross‐centre collaboration set up to foster sharing among peer supporters.

#### 3.3.2 Strategy 2: Fostering Collaboration Through Peer Sharing and Mentorship

Some expressed the importance of consulting senior peer supporters when their supervisors (i.e., social workers) were occupied with workload. This preference for peer‐to‐peer sharing highlighted the trust and appreciation among older adults. Fostering such a mutual learning and mentoring culture may also create a more sustainable mentorship cycle for skill transfer in the long run:

We can also reach out to a more experienced mentor for advice. This way, if any issues arise, we can turn to the mentor instead of constantly trying to find a social worker, who might be very busy. The mentor can help guide the less experienced Peer Supporters in their roles, which makes them feel more comfortable knowing they have someone senior to turn to for support. Everyone appreciates having a mentor, especially when they're new and looking for guidance. And as they gain more experience, they can then pass on that support by mentoring the newcomers in return.

PS02, Female, Age 59

A senior peer supporter also shared her attitude towards knowledge inheritance:

Now, “the Yangtze River's waves behind push on those ahead” (the younger generation is replacing the old). Once you teach them, they can take charge. […] I will also try to share what I have learned and see if it works.

PS03, Female, Age 62

Further, a junior peer supporter expressed appreciation and work efficacy brought from the real‐life practice of the seniors:

It's really great. Like, I would know who to ask and what to ask. I naturally brought it up. When it came to admin‐level tasks, or if I didn't know how to fill in the working hours—which the social workers hadn't taught me—that's when the senior Peer Supporters stepped in.

PS01, Female, Age 52

This mentorship could also encourage peer supporters from different generations (i.e., 50–59, 60–69, 70–79 and ≥ 80 years) to exchange knowledge in service provision. Another senior peer supporter noted:

I really think the younger peer supporters are fantastic with technology. They have a genuine knack for computer‐related tasks. […] They can help with data entry or manage the tech side of things during group sessions—they're truly amazing at that! On the other hand, we might not be as comfortable in those areas. […] Well, I can share insights on handling difficult situations. For example, there are times when they go to visit someone, and the person doesn't answer the door. They encounter challenges like that, and I can help them navigate those experiences.

PS23, Female, Age 70

#### 3.3.3 Strategy 3: Creating a Safe and User‐Centred Environment

Establishing a safe and trustworthy environment for service users was also considered crucial, especially when working with those of the opposite sex. One peer supporter suggested that the first meeting be held in the centre:

When it comes to interacting with the opposite sex, visiting them at home can sometimes lead to complications. Neighbours might see us, and it could create a negative impression. That's why I usually invite them to meet at the centre instead. We have plenty of spaces and meeting rooms there where we can have a good conversation. I've also noticed that bringing them to the centre has an added benefit. Although we were trained for home visits, meeting at the centre aligns better with our ultimate goal of helping them reintegrate into social life.

PS06, Male, Age 60

Another highlighted the importance of initiating conversations based on service users' interests:

I think a great way to kick off a conversation is by sharing some interesting information about a topic they might not know much about. This can really spark their interest and keep the dialogue flowing. For instance, I've found that older men often enjoy discussing history. So, I like to do a bit of research online to learn about important figures and events from their time and political context.

PS13, Female, Age 69

## 4 Discussion

This study examined the challenges experienced by older adults trained to provide peer support services as part of an intervention service for older adults with depressive symptoms, with a primary aim of identifying their self‐developed, real‐world strategies to overcome these difficulties. Our study extended the observations in the previous literature to show that several challenges, such as role ambiguity, difficulties in drawing boundaries, and perceived deficits in skills over time, may also be experienced by peer supporters in the context of late‐life mental health services. By further elucidating the strategies that peer supporters adopt to overcome these implementation challenges, our findings also demonstrated the potential to draw on the wisdom of older adults to improve future service experiences for other peer supporters in the real‐world setting.

Across the various strategies highlighted by peer supporters, the building of more authentic and meaningful engagements with users, as well as the fostering of collaboration among peers and with professionals, emerged as recurrent themes. Whereas previous work has often highlighted the underpaid and non‐professionally recognised nature of peer supporters as major concerns [16], our findings offered an alternative perspective in showing that peer supporters generally prioritised engaging in socio‐emotionally meaningful service experiences, even if on a voluntary basis.

While prior research on peer support has identified a sense of role confusion arising from a lack of clarity and perception of being replaceable informal staff [16], our peer supporters primarily expressed having mixed feelings about receiving monetary compensation for perceived voluntary behaviours. Interestingly, there have been increasing discussions surrounding the problem of peer support workers being underpaid and undervalued [32]. The provision of regular salaries to older adults for their time given to peer support, as a form of meaningful paid employment, is also in line with the framework of productive ageing. Howver, the lack of consideration for the perspectives of peer supporters could also contribute to a heightened sense of pressure and reduced intrinsic motivation [33]. Clarifying the roles of peer supporters at the outset of training and supervision will thus be crucial. Incorporating the strategy of ‘centring attention on authentic and meaningful engagement with users' may also help overcome the feelings of distress associated with such ambiguities in roles.

Contrary to productive ageing studies that underscore ageism and prejudicial attitudes towards older workers [6, 34], these were not experienced by our peer supporters. Difficulties in disclosing personal mental health experiences and stigmatisation for recovered peer supporters, as previously reported [18, 35], were also not challenges encountered in our sample. It is possible that these discrepancies may stem from the absence of questions related to discrimination or stigma in our semi‐structured interview guide. Another possibility, nevertheless, may relate to the positioning of peer supporters in our present project within a mental health intervention context as trained older adults with similar life experiences, rather than people with a history of mental disorders, which could have fostered a more open and less stigmatising environment for more genuine connections to be built. It may be helpful for future work to examine in greater depth how differences in the definition of peer support workers might contribute to differences in their service provision experiences.

To address uncertainties in communication and peer support skills, continuous mutual learning and knowledge exchange in healthcare settings have been identified as crucial both in our present study and in previous work [18, 36]. Aside from seeking professional support, the peer supporters we interviewed demonstrated foresight in programme development and sustainability by suggesting a mentorship system, wherein senior peer supporters could guide newly trained peer supporters. In contrast to the traditional perspective of older adults as passive recipients of care, promoting such a mentorship programme could empower older adults to exchange personal knowledge and facilitate the building of deeper social connections, possibly fostering a greater sense of autonomy [16]. Such peer support mentorship might also reflect the desire for generativity among experienced older adults during the ageing process [37, 38], which may not only enhance service delivery but can also be beneficial to the psychological well‐being of these older adults [39].

### 4.1 Limitations

Despite the insights gathered from peer supporters in this study, we also note several limitations. Although the JoyAge intervention is currently delivered across all 18 districts of Hong Kong, covering the entire territory, we acknowledge that recruiting peer supporters from a single project could undermine the generalisability of the observations. Since the majority of our peer supporters were female and did not have a history of mental health disorder, our findings may not be directly generalised to peer supporters in more specialised mental healthcare settings, nor to other non‐mental health settings. Nevertheless, given that the challenges raised in peer support services were generally in line with those previously reported [16] and difficulties experienced among the older adult population, the additional insights into the real‐world solutions developed by peer supporters themselves may serve as an important basis for future work in the co‐production of service guidelines in peer support, especially in non‐Western contexts. Despite the interviewees being free from direct service delivery and the emphasis on a free sharing space in the focus groups, there could also have been social desirability biases, wherein participants might have responded in manners that portrayed a more stigma‐free, supportive and capable workplace. We also note the possibility that peer supporters without effective strategies could have resigned from the service, thereby leaving unknown challenges and potential solutions unexplored. Future studies could also expand the sample size and explore additional themes that might emerge from a larger and more diverse population. Examining factors that might contribute to peer supporters leaving the service would also be helpful.

### 4.2 Implications

Apart from autonomy and leadership, the older adults in our study showed resilience in navigating peer support challenges. The present study demonstrated that peer supporters have the capability to leverage their wisdom in balancing the need to cater to service users' interests and needs, as well as professionalism (e.g., drawing clear boundaries), to facilitate their practice in community mental health services. The preference for developing a mentorship system also highlights the potential for peer supporters to take on leadership and supervisory roles in future services. To further foster productive ageing and enhance the mental health workforce, governments may also consider incorporating mental health peer supporters into future aged care services.

Of note, rather than simply providing conventional training designed by professionals, the incorporation of strategies developed by experienced peer supporters as observed in our study (i.e., highlighting the importance of centring attention on authentic and meaningful engagement with users; managing expectations and drawing consensus; building support networks, proactive learning and continuous development; fostering collaboration through peer sharing and mentorship; and creating a safe and user‐centred environment) may be crucial to improve the long‐term sustainability of peer support services. For instance, reinforcing elements of peer support work that older adults value (e.g., volunteering with a meaning, authentic connections and learning peer supporter knowledge) might help reaffirm their commitment to peer support and address the negative psychological experiences caused by possible role ambiguity. Encouraging users to seek external resources (e.g., building community networks for service users) may also help avoid overdependence on peer supporters following service cessation. With previous work demonstrating the feasibility and acceptability of co‐producing training programmes with peer supporters from various settings [40], similar processes may also be applied to future developments of peer support work in mental health and aged care settings. Local stakeholders can actively engage peer supporters to share their experiences within future training programmes and supervision sessions.

## 5 Conclusion

The findings illustrated gaps in existing peer support training programmes, particularly in non‐Western contexts, while highlighting the crucial input from older adults as active agents and co‐designers of peer support services. The proactive problem‐solving strategies adopted by older adults in real‐world settings demonstrate an abundance of valuable manpower in the community who are devoted to providing mental healthcare. To ensure more sustainable implementation of peer support services, clinicians and relevant stakeholders could reference these bottom‐up insights to enhance services in collaboration with older adults. Addressing the challenges faced by peer supporters is also crucial to ensure they are honoured as equal and active partners in the mental health workforce.

## Author Contributions

**Eric Kwok Lun Yiu:** conceptualisation, data curation, methodology, formal analysis, writing – original draft, writing – review and editing. **Stephanie Ming Yin Wong:** conceptualisation, investigation, formal analysis, supervision, project administration, validation, writing – original draft, writing – review and editing. **Jessica Kang Qi Lee:** data curation, methodology, formal analysis, validation, writing – review and editing. **Zuna Loong Yee Ng:** data curation, methodology, formal analysis, validation, writing – review and editing. **Dara Kiu Yi Leung:** project administration, methodology, writing – review and editing. **Wai Chi Chan:** project administration, methodology, writing – review and editing. **Gloria Hoi Yan Wong:** project administration, methodology, writing – review and editing. **Terry Yat Sang Lum:** funding acquisition, resources, project administration, methodology, writing – review and editing.

## Ethics Statement

Ethical approval was granted by the Human Research Ethics Committee (HREC) of the University of Hong Kong.

## Conflicts of Interest

The authors declare no conflicts of interest.

## Supporting information


**Supporting Table S1:** Standards for Reporting Qualitative Research Checklist. **Supporting Table S2:** Semi‐structured interview guide.

## Data Availability

The data and analytical codes used to support the findings of this study are available upon reasonable request to the corresponding author.
