# Supplementary material for: Navigating Challenges in Peer Support Work: Perspectives of Peer Supporters From a Stepped Care Intervention for Older Adults With Depressive Symptoms
Source: Health Expect. 2025 Sep 10;28(5):e70430. doi: 10.1111/hex.70430 (PMC12422112; doi:10.1111/hex.70430)
Supplement: Supplementary file 1 — Supporting Table S1: Standards for Reporting Qualitative Research Checklist. Supporting Table S2: Semi‐structured interview guide. [file HEX-28-e70430-s001.docx]

**Supplementary Material**

**Navigating challenges in peer support work: perspectives of peer supporters from a stepped care intervention for older adults with depressive symptoms**

## Supplementary Table S1. Standards for Reporting Qualitative Research Checklist

|  | **Page/**  **Line no(s).** |
| --- | --- |
| **Title and abstract** |  |
| **Title** - Concise description of the nature and topic of the study. Identifying the study as qualitative or indicating the approach (e.g., ethnography, grounded theory) or data collection methods (e.g., interview, focus group) is recommended | P.1 |
| **Abstract** - Summary of key elements of the study using the abstract format of the intended publication; typically includes background, purpose, methods, results, and conclusions | P.2 |
| **Introduction** |  |
| **Problem formulation** - Description and significance of the problem/phenomenon studied; review of relevant theory and empirical work; problem statement | P.3-4 |
| **Purpose or research questio**n - Purpose of the study and specific objectives or questions | P.4 |
| **Methods** |  |
| **Qualitative approach and research paradigm** - Qualitative approach (e.g., ethnography, grounded theory, case study, phenomenology, narrative research) and guiding theory if appropriate; identifying the research paradigm (e.g., postpositivist, constructivist/ interpretivist) is also recommended; rationale** | P.4 |
| **Researcher characteristics and reflexivity** - Researchers’ characteristics that may influence the research, including personal attributes, qualifications/experience, relationship with participants, assumptions, and/or presuppositions; potential or actual interaction between researchers’ characteristics and the research questions, approach, methods, results, and/or transferability | P.5-6 |
| **Context** - Setting/site and salient contextual factors; rationale** | P.4 |
| **Sampling strategy** - How and why research participants, documents, or events were selected; criteria for deciding when no further sampling was necessary (e.g., sampling saturation); rationale** | P.4-5 |
| **Ethical issues pertaining to human subjects** - Documentation of approval by an appropriate ethics review board and participant consent, or explanation for lack thereof; other confidentiality and data security issues | P.7, P.19 |
| **Data collection methods** - Types of data collected; details of data collection procedures, including (as appropriate) start and stop dates of data collection and analysis, iterative process, triangulation of sources/methods, and modification of procedures in response to evolving study findings; rationale** | P.4–6 |
| **Data collection instruments and technologies** - Description of instruments (e.g., interview guides, questionnaires) and devices (e.g., audio recorders) used for data collection; if/how the instrument(s) changed over the course of the study | P.4–6 |
| **Units of study** - Number and relevant characteristics of participants, documents, or events included in the study; level of participation (could be reported in results) | P.4-5, 7-8 |
| **Data processing** - Methods for processing data prior to and during analysis, including transcription, data entry, data management and security, verification of data integrity, data coding, and anonymization/de-identification of excerpts | P.4–5 |
| **Data analysis** - Process by which inferences, themes, etc., were identified and developed, including the researchers involved in data analysis; usually references a specific paradigm or approach; rationale** | P.6-7 |
| **Techniques to enhance trustworthiness** - Techniques to enhance trustworthiness and credibility of data analysis (e.g., member checking, audit trail, triangulation); rationale** | P.7 |
| **Results/findings** |  |
| **Synthesis and interpretation** - Main findings (e.g., interpretations, inferences, and themes); might include development of a theory or model, or integration with prior research or theory | P.7-16 |
| **Links to empirical data** - Evidence (e.g., quotes, field notes, text excerpts, photographs) to substantiate analytic findings | P.7-16 |
| **Discussion** |  |
| **Integration with prior work, implications, transferability, and contribution(s) to the field -** Short summary of main findings; explanation of how findings and conclusions connect to, support, elaborate on, or challenge conclusions of earlier scholarship; discussion of scope of application/generalizability; identification of unique contribution(s) to scholarship in a discipline or field | P.16-18 |
| **Limitations** - Trustworthiness and limitations of findings | P.17 |
| **Other** |  |
| **Conflicts of interest** - Potential sources of influence or perceived influence on study conduct and conclusions; how these were managed | P.19 |
| **Funding** - Sources of funding and other support; role of funders in data collection, interpretation, and reporting | P.19 |
|  |  |
| *The authors created the SRQR by searching the literature to identify guidelines, reporting standards, and critical appraisal criteria for qualitative research; reviewing the reference lists of retrieved sources; and contacting experts to gain feedback. The SRQR aims to improve the transparency of all aspects of qualitative research by providing clear standards for reporting qualitative research. | |
| **The rationale should briefly discuss the justification for choosing that theory, approach, method, or technique rather than other options available, the assumptions and limitations implicit in those choices, and how those choices influence study conclusions and transferability. As appropriate, the rationale for several items might be discussed together. | |

**Reference:** O’Brien, B. C., Harris, I. B., Beckman, T. J., Reed, D. A., & Cook, D. A. (2014). Standards for reporting qualitative research: a synthesis of recommendations. *Academic Medicine, 89*(9), 1245–1251.

## Supplementary Table S2. Semi-structured interview guide

**Part I: Review of Peer Support Work & Training Programme (10 mins)**

1. How would you describe your relationships with service users and social workers?
2. How would you introduce the role of a Peer Supporter to service users?

**Part II: Service Challenges, Strategies, and Additional Training Support (45 mins)**

1. Since you may have completed your training for some time now, and you all have been actively engaged in peer support services, what challenges have you encountered so far?
   - Follow-up: Most of you rated [this core competency] (E. L. Y. Wong et al. 2024) as your strongest/weakest. Is there any reason behind this?
     *(N.B. These survey responses were collected before the focus groups)*

*(Follow-up on any identified root causes of the difficulties)*

1. How did you address or overcome these challenges?
   - Follow-up: Did you come up with solutions on your own? What made you decide to do this?
2. Do you think the Peer Supporter training has been sufficient in facilitating your service provision?
3. What additional training or other support would be valuable to you?
   *(Potential prompts: ‘one-size-fits-all’ vs. personalised training; core curriculum + electives vs. specialised courses?)*
4. Do you have any suggested additions to the training content that would facilitate your handling of current challenges? Can you share more on the reasons? *(Follow-up on examples and rationales)*

**BREAK (10 mins)**

**Part III: Impact of Lived Experience & Core Competencies (30 mins)**

1. Can you share more on how your recovery experiences have influenced your (i) training participation, and (ii) interactions with service users?

*(Follow-up on any benefits and challenges)*

1. How have your recovery experiences and core competencies shaped your Peer Support work (e.g., in working with service users)?
   - Follow-up: Which of the factors you mentioned carries more weight in your opinion?

**Part IV: Social Contributions of Peer Supporters (30 mins)**

1. As a Peer Supporter, what do you think differentiates you from a regular volunteer after training?
2. What are your thoughts on older adults providing Peer Support services?
   *(Potential prompt: Imagine a group of older adults working in the community service units, what do you feel?)*
   - Follow-up: What do you feel after providing service/handling challenges?
3. What are your thoughts on whether Peer Supporters should be considered a formal occupation?
   - Follow-up: Rationale for remuneration?
4. What are your thoughts on the impact of the Peer Supporter programme on community mental health and Hong Kong’s healthcare ecosystem?
   *(Potential prompt: Why is Peer Support needed in society?)*
   - Follow-up: Potential for incorporating Peer Supporter work into Hong Kong’s healthcare system?
5. Should career progression exist in Peer Support (e.g., ‘senior’ or ‘master’ Peer Supporters)?
   If yes:
   - What would the differences be between a regular and a ‘master’ Peer Supporter?
   - What would the criteria be for a Peer Supporter to progress into a ‘master’ role?
   - Follow-up: Relevance of recovery experience/competencies?
6. Would you feel confident in:
   - Leading interest groups?
   - Conducting training?
   - Designing/managing Peer Supporter programmes?

**Part V: Future Development & Feedback (5 mins)**

**Reference:** Wong, E. L. Y., Yau, J. H. Y., Sze, L. C. Y., Kanagawa, H. S., Leung, D. K. Y., Liu, T., ... & Lum, T. Y. S. (2024). A core competency framework for mental health peer supporters of older adults in a Chinese community: cross-culturally informed Delphi study. *BJPsych Bulletin, 48*(2), 92–99.
